# The Culturable Mycobiome of Mesophotic *Agelas oroides*: Constituents and Changes Following Sponge Transplantation to Shallow Water

**DOI:** 10.3390/jof7070567

**Published:** 2021-07-16

**Authors:** Eyal Ben-Dor Cohen, Micha Ilan, Oded Yarden

**Affiliations:** 1 School of Zoology, George S Wise Faculty of Life Sciences, Tel Aviv University, Tel Aviv 6997801, Israel; eyalbendor@gmail.com (E.B.-D.C.); milan@tauex.tau.ac.il (M.I.); 2 Department of Plant Pathology and Microbiology, The RH Smith Faculty of Agriculture, Food and Environment, The Hebrew University of Jerusalem, Rehovot 7610001, Israel

**Keywords:** mycobiome, marine sponge, marine fungi, *Agelas*, mesophotic

## Abstract

Marine sponges harbor a diverse array of microorganisms and the composition of the microbial community has been suggested to be linked to holo-biont health. Most of the attention concerning sponge mycobiomes has been given to sponges present in shallow depths. Here, we describe the presence of 146 culturable mycobiome taxa isolated from mesophotic niche (100 m depth)-inhabiting samples of *Agelas oroides*, in the Mediterranean Sea. We identify some potential in vitro interactions between several *A. oroides*-associated fungi and show that sponge meso-hyl extract, but not its predominantly collagen-rich part, is sufficient to support hyphal growth. We demonstrate that changes in the diversity of culturable mycobiome constituents occur following sponge transplantation from its original mesophotic habitat to shallow (10 m) waters, where historically (60 years ago) this species was found. We conclude that among the 30 fungal genera identified as associated with *A. oroides*, *Aspergillus*, *Penicillium* and *Trichoderma* constitute the core mycobiome of *A. oroides,* and that they persist even when the sponge is transplanted to a suboptimal environment, indicative of the presence of constant, as well as dynamic, components of the sponge mycobiome. Other genera seemed more depth-related and appeared or disappeared upon host’s transfer from 100 to 10 m.

## 1. Introduction

Sponges (phylum Porifera) are a diverse and abundant filter-feeding phylum of sessile marine invertebrates, with close to 10,000 known species [1]. They inhabit hard and soft bottom habitats, while a few species (ca. 150) occupy fresh water [2]. Sponges are an important component of the benthic fauna, with a significant impact on biogeochemical cycling of key nutrients [3]. In some ecosystems sponges provide three-dimensional habitats in the benthic-exposed environment [4]. Thus, they influence the composition, diversity, and abundance of the epibenthic faunal community [5] and the pelagic fauna [6]. Marine sponges are known to harbor a large diversity of microorganisms [7,8,9]. These associated microorganisms are thought to be involved in a variety of ecological functions including production of secondary metabolites [10,11]. While experimental studies suggest that the composition of the microbial community is linked to holo-biont health, the potential role of the microbiome as involved in sponge acclimation and adaptation to environmental change is unknown [3]. While sponges host microorganisms from all domains of life [12], sponge-associated eukaryotes have been the most recent to gain attention. These, and in particular, fungi, have been shown to be both common and diverse in almost every class of sponges [11,13,14,15,16,17,18]. While host genetics, environmental factors and geography have been shown to influence the sponge prokaryotic microbiome [19,20], the influence of these factors on the mycobiome have yet to be characterized.

*Agelas oroides* (Demospongiae) is a massive sponge, with size ranges between 5 cm to 25 cm. It is well known for its secondary metabolites production [21,22,23]. It can be found throughout the Mediterranean Sea, along a depth gradient of 4–115 m, indicating that this sponge can inhabit mesophotic niches. Over the last decade, mesophotic coral ecosystems (MCEs), found at ocean depths of 30–150 m, have gained increasing levels of attention [24,25,26]. Typically, this niche is characterized by lower temperatures and light intensities than the shallow water, and is an environment richer in nutrients. This habitat has been considered to be part of the “deep reef refuge hypothesis”, which suggests that, following some adjustment, macro-organisms inhabiting this niche can find refuge from biotic and a-biotic stresses common in shallower habitats [27]. In spite of its reported broad depth range, *A. oroides* was last recorded in Israeli Levant region in shallow waters (<7 m) 50 years ago [28], and since then considered as lost to this region. It was recently re-found as flourishing along the Israeli coastline, at mesophotic (ca. 100 m) depths [23,29]. A recent transplantation experiment supports the suggestion that *A. oroides* disappeared from the Levant shallow water due to the increase in shallow water summer temperatures, and the extended period of these higher water temperatures during the past 50 years [29].

Members of the genus *Agelas* have been described as high microbial abundance (HMA) sponges [30]. One suggested role for the large microbial consortia present in these sponges is in nutritional supplementation [31]. The number of reports on *Agelas*-associated fungi is limited and has mainly focused on several *Aspergillus* spp. that have been isolated from *A. oroides* and their capacity to produce natural products [32,33].

Here, we report on culturable constituents of the mesophotic *A. oroides* mycobiome, identify some potential interactions between several *A. oroides*-associated fungi and describe changes in mycobiome constituents following sponge transplantation from their mesophotic habitat to shallow (10 m) waters, where historically this species was found.

## 2. Materials and Methods

### 2.1. Sponge Sample Collection

**Figure 1:**
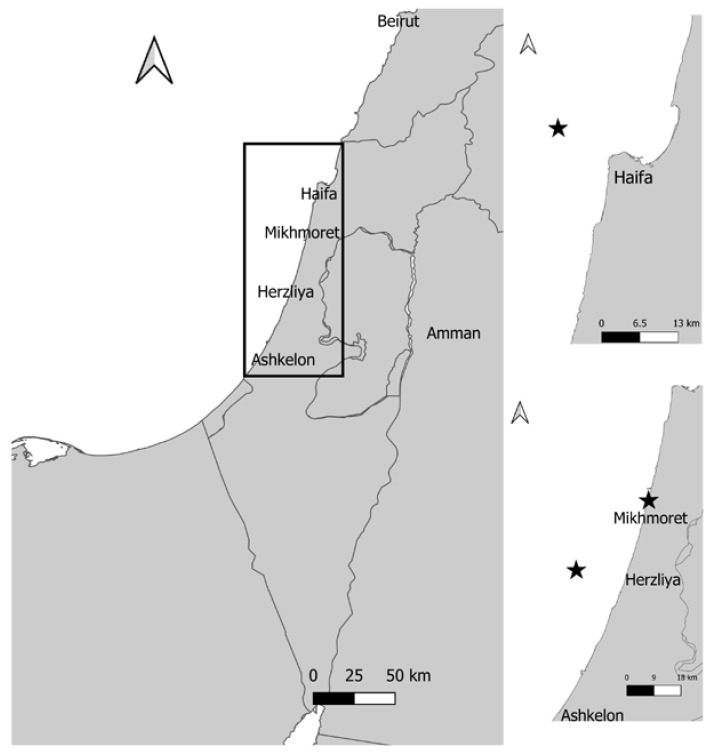
Map of *Agelas oroides* sampling and transplantation points (marked by asterisks).

Samples (n = 10) of the sponge *Agelas oroides* were collected (with a permit from the Israel Nature and Parks Authority) from the Mediterranean Sea by a remotely operated vehicle (ROV) at ~100 m depth from two sites, using the R/V Mediterranean Explorer (EcoOcean) (Figure 1), as described in detail by Idan et al. [29]. Two samples (A, B) were collected from Haifa Rosh HaCarmel (32°52.44′ N, 34°51.47′ E) and eight samples (C–J) were collected approximately 16 km off-shore at Herzliya, Israel (32°10.62′ N, 34°37.98′ E). Sponge samples A and B were collected in the autumn (September 2017), C-G during the winter (January 2018), H in the summer (July 2018) and samples I and J also in the winter (February 2019). The approximate size of each sponge sample was ~350 cm^3^. Samples were kept in sealed plastic containers with seawater and treated in the laboratory within three hours post collection. Using a sterile blade, the sponges were cut in the center to expose inner parts. These core sponge sections (~5.0 cm^3^) were dipped twice in sterile double distilled water (SDDW) prior to plating on fungal growth media.

For the transplantation experiment, two sponge samples (~430 cm^3^) were collected from the mesophotic habitat using the ROV. The samples were maintained, for two weeks, in open water tables at the marine facility of Ruppin Academic Center (Mikhmoret). Then, the sponges were stabilized by being attached to a brick (400 cm^2^) and placed (by SCUBA) in a cage at 10 m depth in the Mediterranean Sea (“Playground” site, Mikhmoret beach, 32°25.71′ N, 34°.52.39′ E). The cage was covered with a plastic net (3 cm mesh) to prevent animals from feeding on the sponges and to reduce light intensity. Every two weeks three sponge fragments (~5.0 cm^3^) were collected from different parts of the sponges, and determined, on the basis of visual inspection, as either healthy, visibly unhealthy or dead. The fragments were compressed and fungal isolation and identification were carried out as described below.

### 2.2. Isolation and Identification of Agelas oroides-Derived Fungi

After rinsing the sponge core sections, they were either compressed using an autoclaved mortar and pestle, as previously described [17] or cut to smaller fragments. Following the compression procedure, 100 µL of sponge extract were plated on potato dextrose agar (PDA, Difco, Franklin Lakes, NJ, USA) Petri dishes amended with 250 mg/L chloramphenicol. Petri dishes were incubated at 25 °C, in the dark, until a mycelium emerged (3–60 days). Following fungal growth, disks (0.4 cm diameter) from the edge of the colony were transferred to fresh medium, until pure cultures were obtained. In parallel, the smaller, not compressed, sponge fragments were placed on identical, slanted, medium in 13 mm test tubes until fungal growth was evident. Pure cultures from these tubes were purified as described above.

To increase the variety of isolated fungi, sponge samples were plated on additional dishes, amended with either cycloheximide (500 µg mL^−1^, Sigma, St. Louis, MO, USA), the benzimidazole fungicide Benomyl (10 µg mL^−1^, Dupont, Wilmington, NC, USA) or the pyrimidine sulfamate fungicide Bupirimate (10 µg mL^−1^, Makhteshim-Agan Group, Airport City, Israel), as previously described by Paz et al. [17].

For DNA-based molecular identification of the isolated fungi, fungal mycelium was scraped from the culture plates and suspended in 2 mL tubes containing 200 µL de-ionized water (NANOpure, Barnstead Co., Newton, MA, USA) and an equal amount of 0.5 mm glass beads (acid washed, Sigma-Aldrich, St. Louis, MO, USA). The tube was agitated using a Mini Bead Beater (Biospec Products Inc., Bartlesville, OK, USA) for 100 s, followed by 10 min boiling to inactivate endogenous nucleases. The samples were cooled to room temperature and subsequent DNA isolation was carried out using the GenElute™ Plant Genomic DNA miniprep kit (Merck, Herzliya Pituach, Israel) as described in the user manual.

PCR was carried out with S1000™ Thermal Cycler (Bio-Rad, Hercules, CA, USA) using PCRBIO VeriFi mix (PCR BIOSYSTEMS, Wayne, PA, USA). Primers used in this study are listed in Table 1. These included internal transcribed sequences of rDNA, as well as specific partial gene sequences (on the basis of different genera) used to identify species. Initial denaturation of DNA was carried out at 95 °C for 1 min followed by 35 cycles of three-step PCR amplifications consisting of denaturation at 95 °C for 0.5 min, primer annealing at the appropriate temperature (Table 1) for 15 s, and extension at 72 °C for 30 s. PCR products were separated by agarose gel electrophoresis. Amplicons were purified (Wizard SV Gel and PCR Clean Up System, Promega, San Luis Obispo, CA, USA), sequenced (MacLab, South San Francisco, CA, USA) and assembled with the Fragment Merger Tool [34]. Fungal strain identification was based on BLAST comparison of the sequences obtained with available databases. 

### 2.3. Fungal-Fungal Interactions and Assays of Growth on Sponge Tissue

Interactions between three of the strains isolated from mesophotic sponge samples (*Alternaria alternata* strain CP-02-2; accession number MZ568118; *Parengydontium album* strain CP-03-1; accession number MZ568223 and *Zygosporium masonii* (strain CP-03-2; accession number MZ568332) were analyzed in dual cultures. Agar plugs (4 mm diameter) from the edges of growing colonies were placed either in the center (single cultures) or near the periphery (dual cultures) of PDA dishes and incubated at 25 °C for periods ranging 9–18 days, until colony fronts were close enough to each other to visualize the presence or absence of an interaction. To determine growth rates, images of the various colonies (n = 4) obtained at different time points were analyzed using ImageJ [35]. Two way-ANOVA statistical analysis was used to determine the significance of differences in the growth rates.

To determine the temperature-dependent growth rates of *Penicillium stekii* (strain EP-14-1; accession number MZ568306), mycelial plugs (4 mm) of the fungus were first placed in the center of an SWA-containing petri dish. The dishes were incubated at either 18 °C, 25 °C, 28 °C, 31 °C or 34 °C. The temperatures in the mesophotic habitat were stable between January to October 2018 (19.5 °C–18.1 °C) while the temperatures in the shallow water ranged between 17.5 °C–31 °C. Hence, the temperatures tested represented temperatures prevalent in the Mediterranean Sea (excluding 34 °C, which is higher than previously measured temperatures; based on Idan et al. [29]). Growth rates were measured (n = 4) as described in the dual culture experiments.

In order to examine if *A. oroides* sponge fragments could support fungal growth, one specimen of *A. oroides* was collected from the mesophotic habitat and divided into two. One part was compressed (“pressed sponge”) and the other did not undergo any mechanical treatment (“native sponge”). Both subsamples were autoclaved and placed on pre-moistened Whatman filter paper in standard petri dishes. Disks (3 mm) of *P. album* cultured on SWA were placed on the sponge subsamples (two replicates each, of pressed and native sponge samples). After seven days at 18 °C, fungal growth was imaged using a Stemi SV 6 stereoscope (Zeiss, Oberkochen, Germany). A follow-up experiment, using the same samples, included adding 200 µL of Potato dextrose broth (PDB) on one side of the pressed sponge and observing fungal growth at various time points after the PDB amendment.

## 3. Results

### 3.1. Fungal Diversity in Agelas oroides

**Figure 2:**
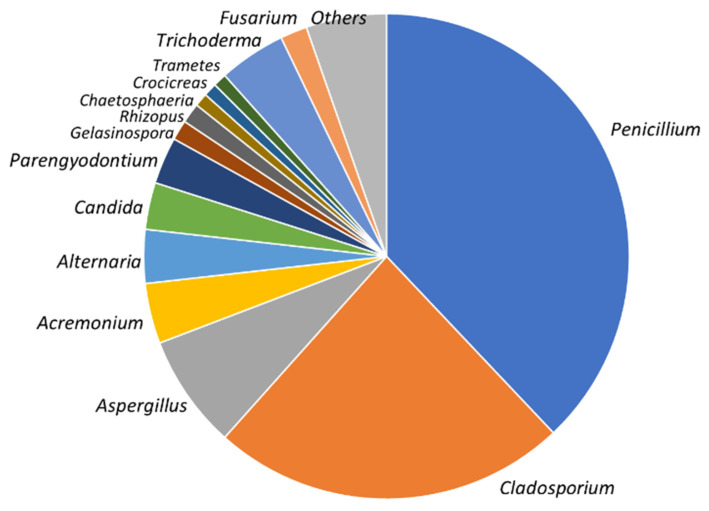
Relative abundance of culturable fungal genera isolated from *Agelas oroides*.

**Figure 3:**
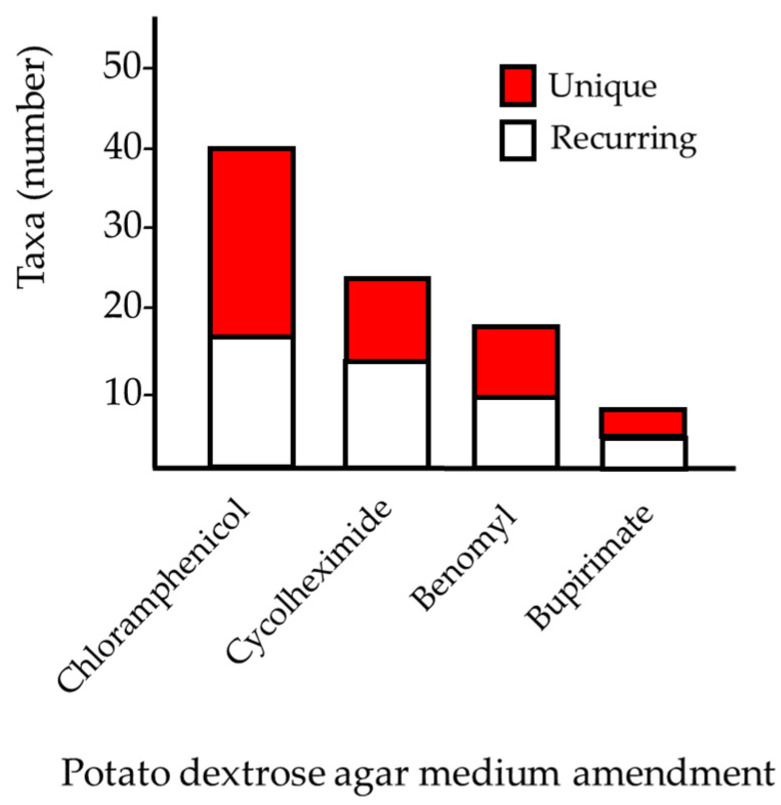
The number of unique and recurring fungal taxa observed on PDA medium with different amendments.

Overall, 146 taxa, represented by 237 sponge-derived culturable fungal colonies, were isolated from the 10 *A. oroides* specimens originally collected from the mesophotic sites (Figure 2). Among the cultivated strains, 96% (230 taxa) were identified as Ascomycota (Table 2). Approximately half of them were designated as belonging to four orders (Euritales, Capnodiales, Hypocreales and Pleosporales). Overall, 30 genera were identified on the basis of ITS1 sequencing and a majority of the species comprising the culturable mycobiome were identified using additional molecular markers (Table 1 and Table 2). The most common genera were *Penicillium*, *Cladosporium* and *Aspergillus* spp. (33%, 22% and 11%, respectively) and were present in most of the sponges collected from both mesophotic sites. Amending the isolation medium with cyclohexmide, Benlate or Bupirimate increased the diversity of the obtained taxa. This included 11, 10 and 4 taxa, respectively, which were not isolated on the standard, chloramphenicol-amended, PDA medium (Figure 3). While most identified taxa were found to reoccur in the different mesophotic sponge samples, members of one genus, *Fusarium* spp. were found to occur uniquely in sponge specimens following their transplantation to shallow water (see below).

### 3.2. Members of the Agelas oroides Mycobiome Exhibit a Variety of Fungus-Fungus Interactions

**Figure 4:**
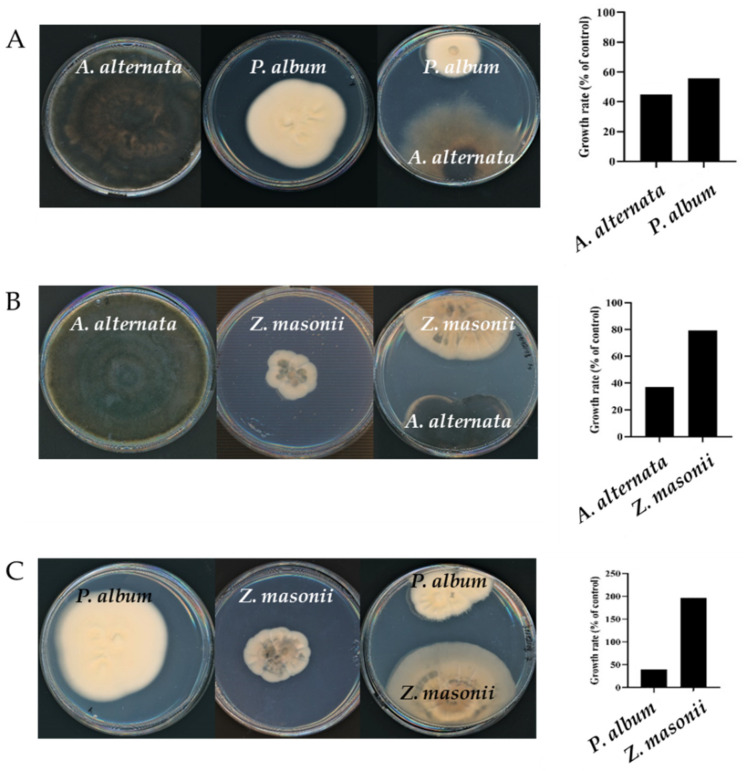
Growth of the *A. oroides* mycobiome constituents *Parengyodontium album, Alternaria alternata and Zygosporium masonii* in single and dual cultures. Pictures and measurements were carried out 12, 9 and 18 days after inoculation, for panels (**A**–**C**), respectively. Within each panel, differences in growth rates are significantly different (*p* ≤ 0.01).

A variety of possible interactions can be anticipated to occur between fungi occupying the same niche. To identify some of these potential interactions, a dual culture approach was used. When *Alternaria alternata* and *Parengyodontium album* were cultured together, the colony area of the two fungi was 55% and 44%, respectively, of that measured of the two species when grown in monoculture under the same conditions (12 days at 25 °C). Morphological changes were also evident in the *A. alternata* colonies. Not only was the colony size smaller and not circular, but the hyphae’s color was lighter, suggesting less melanin was produced. When *A. alternata* was cultured with *Zygosporium masonii*, which is a much less reoccurring member of the culturable *A. oroides* mycobiome, the growth rate of the former was suppressed by about 60% while that of *Z. masonii* was not significantly affected (Figure 4). In contrast to when cultured in the presence of *P. album*, no marked changes in colony morphology were observed. We also examined the outcome of co-culturing *P. album* and *Z. masonii*. In this case it appears that the two species have inverse effects on each other. While growth of *P. album* in the dual culture decreased by about 40%, that of *Z. masonii* increased by almost two-fold when cultured in the presence of *P. album*. Taken together, it appears that diverse potential interactions can occur between members of the *A. oroides* mycobiome, as is evident even in this relatively simple case, comprised of an interaction matrix of based on co-culturing only two species out of three.

### 3.3. Sponge Holo-Biont Constituents Can Support Growth of Parengyodontium album

To determine whether fungi isolated from *A. oroides* have the potential to grow and reproduce under nutritional conditions prevalent in the sponge environment, we cultured five of the most reoccurring species (*Alternaria alternata, Cladosporium halotolerans, Parengyodontium album, Penicillium steckii* and *Zygosporium masonii*) on seawater agar. We were able to clearly observe all stages of their anamorphic life cycle on this low nutrient, high salinity medium. The sensitivity of three of the mentioned species, *C. halotolerans*, *P. album* and *P. steckii* to various temperatures (18–31 °C) which are typical of both mesophotic and shallow depths of the Eastern Mediterranean Sea, was also examined. The optimal temperature for *P. steckii* growth was 18 °C, while *C. halotolerans* and *P. album* exhibited faster growth at moderate temperatures (25 °C and 28 °C). All three fungi exhibited negligible growth at 31 °C and no observable radial growth at 34 °C.

**Figure 5:**
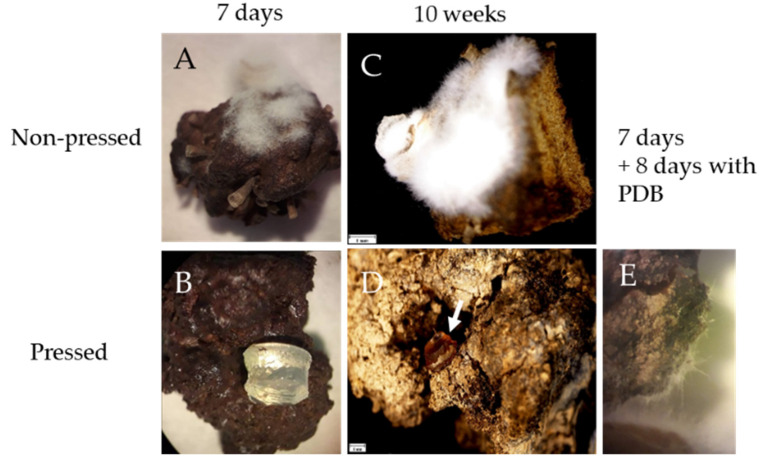
Growth of *Parengyodontium album* on *Agelas oroides*. Mycelial plugs of the fungus on non-pressed (**A**) and pressed (**B**) fragments of *A. oroides*, seven days post inoculation and 10 weeks post inoculation (**C**,**D)** (Plug is marked with arrow). Growth of the *P. album* on a pressed fragment of *A. oroides* 15 days post inoculation (**E**). Seven days after inoculation, potato dextrose broth (PDB) was added to the edge of the sponge.

To determine whether the sponge can serve as a sufficient source of nutrients to support the growth of a mycobiome constituent, we cultured *P. album*, known to be a high producer of extracellular proteases [36], on sterile, intact, *A. oroides* collagen-rich tissue fragments. Discs of cultures grown on sea water agar were placed on either autoclaved sponge cubes or cubes that had been depleted (by applying mechanical pressure) of a significant part of their natural dissolved and water-suspended contents. After seven days of incubation at 18°C, growth was clearly seen on the autoclaved sponge fragments while no observable growth was seen on a similar fragment that was also subjected to pressing (Figure 5A,B). 71 days post inoculation, fungal growth was still observed on the autoclaved (yet otherwise unprocessed) sponge fragment, while no observable growth was detected on the pressed sponge sample (Figure 5C,D). To evaluate if the lack of growth could be due to the possible nutrient-depleted state of the pressed sponge sample, 200 µL of growth medium (PDB) were added to one side of a pressed sponge fragment, one week post-inoculation. Eight days after adding this additional source of nutrients, slight, but clearly observable, fungal growth was evident (Figure 5E). Based on these results, we concluded that while sponge collagen was not sufficient to support growth of *P. album*, the sponge holo-biont contains a sufficient source of nutrients to support growth of this *A. oroides*-associated fungus.

### 3.4. Transfer of Agelas oroides from Mesophotic to Shallow Water Confers Changes in the Mycobiome

**Figure 6:**
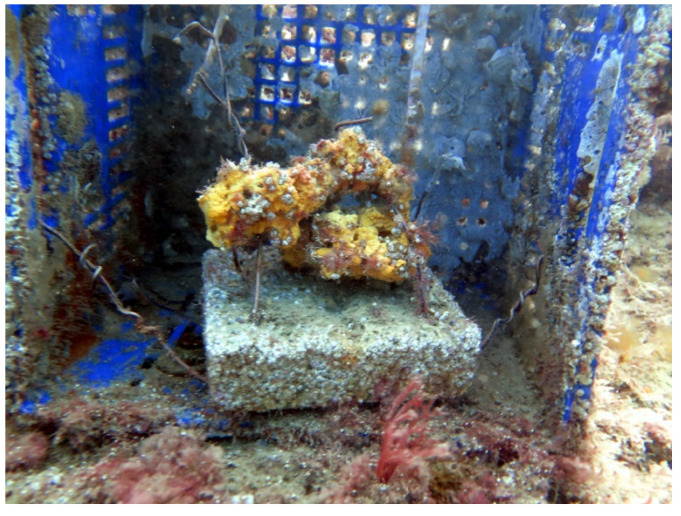
Transplanted *A. oroides*. The sponge was isolated from its mesophotic habitat, secured to a base and transferred to a protected surrounding in shallow water. Concrete base length is 18 cm.

To examine the possible effects of environmental changes on the mycobiome of *A. oroides*, a transplantation experiment was conducted. First, two specimens (I, J) were collected from the mesophotic depth and small samples were cut and used to identify the culturable mycobiome, as part of the experiment described above. The remainder of specimens I and J was placed in in an open seawater system for two weeks and subsequently transferred to a location at 10 m depth, off Mikhmoret beach (Figure 1 and Figure 6). Two weeks later, three fragments from specimens I and J, exhibiting either apparently healthy, unhealthy or dead morphological characteristics, were collected.

**Figure 7:**
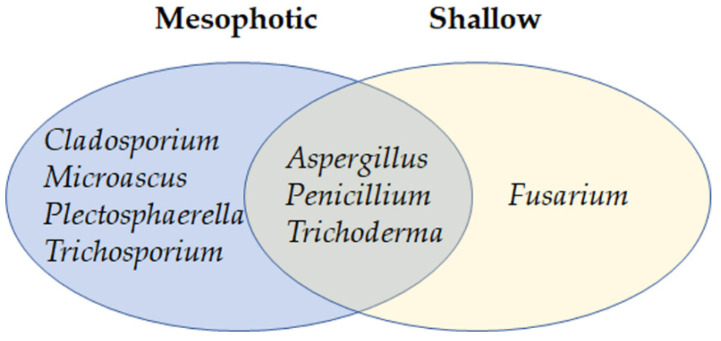
Presence of eight culturable fungal genera in specimens of *Agelas oroides* obtained from mesophotic depth (blue) and transplanted to shallow (10 m) depth.

**Figure 8:**
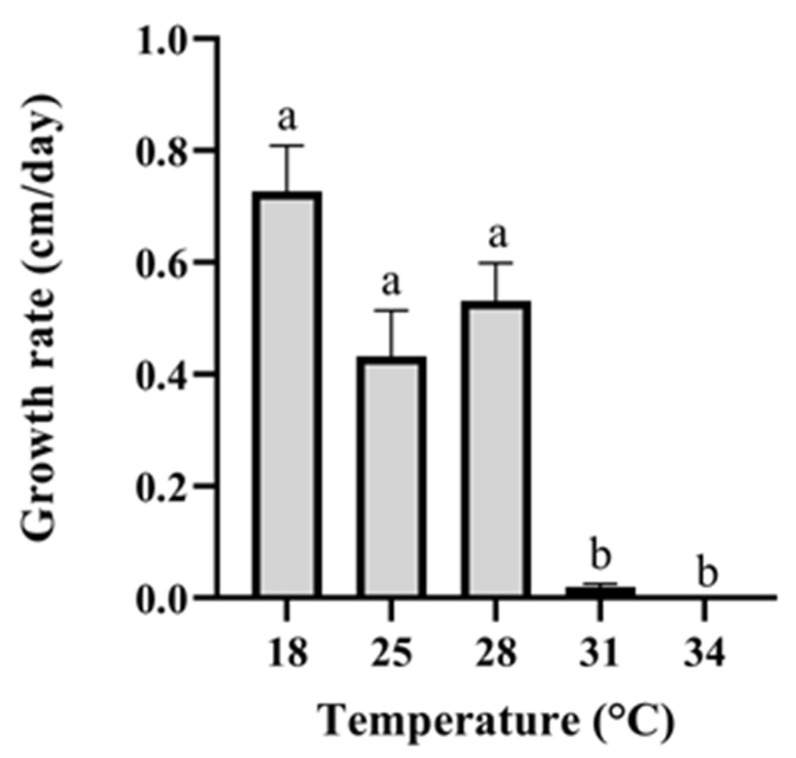
Temperature-dependent growth rate of *Penicillium stekii* isolated from mesophotic *Agelas oroides*. Bars indicate standard error. Different letters above the columns designate the values presented are significantly different (*p* ≤ 0.05).

Two months post transplantation, one specimen had died and was taken for fungal strain isolation and identification. The other survived until mid-July (four months after transplantation). Strains were isolated from specimens I and J on four occasions: initially when they were mesophotic, later when they were apparently healthy in the shallow water, then when they became sick and later dead. The 30 isolated strains from these sponges were comprised of eight genera (Figure 7). The results indicated that the differences in strain abundance were due to the different sea depth source, and not to the health state of the sponge. Among the fungi isolated from sponge samples at the shallow depth, *Aspergillus* (two strains) and *Penicillium* spp. (one strain) were found in both apparently unhealthy, as well as dead, sponge specimens. *Trichoderma* (six strains) and *Fusarium* spp. (six strains) were isolated from apparently healthy, as well as dead, samples obtained from the same depth. The *Fusarium* spp. colonies (Table 2) were the only fungi isolated here that were not found in any of the mesophotic samples throughout this study. Conversely, one of the most common species isolated from sponges obtained from the mesophotic depth, *P. stekii* (47 strains), was not found in any of the transplanted sponge samples. As one of the most distinct differences between the mesophotic and shallow depths is the temperature, we hypothesized that the higher temperatures prevalent in the shallow water may have affected *P. stekii*. We therefore measured growth of *P. stekii* isolated from the mesophotic sponge samples at different temperatures. Indeed, in spite of the fungus’ ability to grow well at temperatures ranging from 18–28 °C, hardly any growth was observed at 31 °C and above (Figure 8), suggesting a possible link between temperature and the presence of *P. stekii* in these sponge samples.

## 4. Discussion

Fungi have been repeatedly cultured from a wide variety of sessile marine animals [18]. There has been at least one report of the isolation of a sponge-associated fungus, from *Acanthella cavernosa,* collected from a mesophotic depth [37]. In the case of *Agelas* sp., fungi have been, so far, isolated or identified by DDGE analysis from specimens obtained from shallow depths [33,38,39]. In order to determine whether fungi also thrive in *A. oroides* present in mesophotic depths, we sampled mesophotic sponges and analyzed for the presence of culturable fungi. Overall, we isolated 296 fungal colonies from ten *A. oroides* specimens (Table 2). The genera of 237 sponge-derived isolates were determined on the basis of their internal transcribed rRNA gene spacer. Among the cultivated strains, 96% (230 taxa) were identified as Ascomycota and found to be comprised of 30 genera. Paz et al. [17], who studied the fungal community in another Mediterranean sponge, *Ircinia variabilis*, albeit at a shallow depth, also found the presence of representatives of the same four predominant orders: Capnodiales, Eurotiales, Hypocreales and Pleosporales. The high prevalence of Ascomycota found here is in line with previous studies, using either culture-dependent or metagenomic-based approaches [13,40,41,42]. Fungicide amendments to the isolation media proved advantageous in obtaining a higher diversity of fungal taxa from the sponge host, as has been the case with other sponge species [17,43].

Members of three genera were repeatedly found associated with samples of *A. oroides* (including after host transplantation to shallow water): *Aspergillus*, *Penicillium* and *Trichoderma.* We therefore concluded that, at least within the geographical area analyzed, these comprise the core culturable mycobiome of *A. oroides*. Nonetheless, these genera are ubiquitous in marine environments and thus caution should be used prior to assigning attributes of specificity to the observed associations. In the case of *Trichoderma* spp., this is the first case of identifying a member of this genus in association with *A. oroides*, even though strains of *Trichoderma*, including a new species, *T. beinartii*, [44], have been found in *Iricinia variabilis* (formerly *Psammocinia* sp.), collected from shallow water along the coast of Israel [45]. In addition to their omnipresent nature in association with marine life forms, many strains belonging to the three mentioned genera have been shown to produce bioactive secondary metabolites [11], including in Mediterranean sponges [10,46,47,48]. It is likely that this is also the case with some of the strains isolated from the mesophotic *A. oroides* (and perhaps involved in some of the antagonist fungal–fungal interactions observed in this study). The fact that at least some representatives of the three common genera, *Aspergillus*, *Penicillium* and *Trichoderma* (Figure 7) have been shown to exhibit myco-parasitic capabilities [17,49,50,51] may be indicative of the potential presence of such interactions among the sponge mycobiont constituents. At least in the case of sponge-derived *Trichoderma* spp. some of the strains isolated from *I. variabilis* have been shown to have myco-parasitic capabilities against other fungi isolated from the same sponge [17]. In this study, *Fusarium* spp. were found only in sponges that had been transplanted to shallow water. This genus is extremely diverse [52] and has been shown to be ubiquitous in terrestrial and marine habitats. The ecological roles of the species identified here have yet to be determined, yet one possibility is that these function as post-transplantation acquired saprobes that feed on decaying sponge tissue. Many *Aspergillus* species are known as animal pathogens [53,54,55] and strains of some of those species (e.g., *A. sydowii*, *A. fumigatiaffinis*, *A. novoparasiticus* and *A. flavus*) were isolated during this study. While, to the best of our knowledge, fungal pathogens of marine sponges have yet to be described, the possibility of a sponge being a symptomless vector of a fungal pathogen has been previously suggested [18,56] and it is possible that, in this case too, *A. oroides* may harbor potential fungal pathogens of sponges or other marine animals. *Penicillium* spp. were also among the most abundant fungal species isolated (85 strains). More than half (47 strains) were identified as *P. steckii*. This species has been repeatedly found in the marine environment and in association with a variety of organisms, including tunicates, molluscs, fish, algae and sponges [57,58]. While terrestrial *P. steckii* has been reported to favor higher temperatures (optimal growth at 30 °C; [59]), here, we have found that the sponge-associated. *P. steckii* exhibits maximal growth at 18 °C–28 °C, which are the prevalent temperatures at the mesophotic depth from where the host was obtained (Figure 8). This observed preference of lower temperatures may have, along with other factors, contributed to the fact that this species was not isolated from the sponges that had undergone transplantation to the shallow habitat. It also implies adaptation of this specific strain’s life to the mesophotic habitat, and raises the question of which adaptations would be advantageous to fungi residing in this ecological niche. Interestingly, the fungus did not last long in the sponges when they were transplanted to shallower (10 m) depth. The hosting sponge, however, survived for several months and exhibited characteristic signs of vitality (e.g., ectosome intact, open oscula, and pumping water), including the rebuilding of their body wall and detaching of the necrotic parts which were a result of the transplantation process. However, once ambient temperatures exceeded 28°C indications of stress were evident and, subsequently, all the hosts died [29]. To what extent the observed differences in the sponge mycobiome are cause or consequence has yet to be determined, but at least in the case of *P. stekii*, temperature may well have had an effect on its presence. Differences in the mycobiome at different depths can be expected. In fact, the stability of core mycobiome constituents on the one hand, and changes in non-core components on the other, even along a much smaller difference in depths, has been observed in another sessile marine animal—*Acropora lorites*, even without transplantation [60]. Seven strains of *P. album* (formerly *Engyodontium album*), noted for its production of proteases and cytotoxic secondary metabolites [61,62,63], were isolated from the different *A. oroides* samples. To assess the possible relations between *A. oroides* and *P. album*, a sterilized sponge fragment was inoculated with the fungus. Two months post-inoculation the fungus covered most of the fragment. The fact that *P. album* can grow at 18 °C and has the ability to utilize the sponge meso-hyl (inner section) as a sole source of nutrients, supports the possibility that, within *A. oroides, P. album* potentially has sufficient nutrients to support growth in the mesophotic niche. However, to date, there have been no reports on the presence of hyphae developing in sponges, and this study is no exception.

Typically, sponges are considered to harbor a higher complexity of microbial diversity than corals, but that diversity is more stable [64]. While the nature of fungal survival, growth and reproduction within the living sponge remains to be elucidated, the fact that fungi are so prevalent in marine sponges suggests that they may interact while occupying this niche, as part of the nature of maintaining a stable mycobiome. Furthermore, analysis of sponge microbiomes among *Ircinia* spp. specimens exhibiting different growth forms indicate the presence of stable associations between host sponges and their microbiomes, and perhaps that they potentially contribute to ecological divergence among *Ircinia* species [65]. It is also likely that interactions between mycobiome constituents are part of the dynamics of microbiome stability. Even though only a limited number of fungal strains were analyzed here, a range of interactions from growth suppression to enhanced colony growth was easily detected (Figure 4). Whether or not such interactions occur within the highly complex holo-biont, remains to be determined, it is likely that the physical proximity of multiple microorganisms is accompanied by a plethora of potential outcomes, some of which were observed here. These would also likely include interactions with bacteria and the host itself.

The results obtained in this study indicate the presence of a diverse mycobiome, which includes core members, that can even be cultured from small sponge fragments. It is highly probable that a significant number of additional taxa are part of the sponge mycobiome (core, as well as transient community members). Some are expected to be difficult to culture under the conditions used here, and some may even be obligate biotrophs (on the sponge itself or other members of the holo-biont). As the current consensus on the definition of marine fungi is based on ecological, rather than a taxonomical, basis [18], we have considered all the culturable fungi described here as part of the sponge holo-biont, regardless of the nature of their symbiosis. Considering that the sponge is a filter-feeder, small particles (~0.1 µm), including fungal spores, can be readily found in the sponge. Kumala et al. [66] described osculum dynamics and its effect on filtering rate, as part of the mechanism by which sponges can control their filter feeding parts and clean them. Even given the sponges’ impressive capabilities to digest or extrude particles, fungal propagules can be easily isolated from sponges, indicative of their persistence. As the fungi described here are, for the most part aerobic, availability of oxygen may be a crucial factor to their survival and proliferation within the holo-biont. The ability of the sponge to modulate aerobic and anaerobic metabolism via regulation of its water flow may be one of the factors that determines the fate of its mycobiome [67,68]. It is tempting to speculate that the extent of fungal strain sensitivity to anoxia may play an important role in special and temporal fungal presence and activity within the sponge. Coupling sponge sectioning along genomic and transcriptomic analyses may provide some additional answers to the points raised here and may be instrumental in linking mycobiome residency with mycobiome function within marine sponges.

## Figures and Tables

**Table 1:** Primers used in this study.

Gene Name and Targeted Templates	Primer Designation (Annealing Temp.)	Sequence
Internal Transcribed Spacer (ITS) (all fungal templates)	ITS-5F ITS-4R (62 °C)	GGAAGTAAAAGTCGTAACAAGG TCCTCCGCTTATTGATATGC
ACTINa (ACT) (*Cladosporium* spp.)	ACT-512F ACT-783R (60 °C)	ATGTGCAAGGCCGGTTTCGC TACGAGTCCTTCTGGCCCAT
β-tubulin (Tub2) (*Acremonium* spp.; *Chaetomium* sp.; *Nigrospora* sp.; *Parengydontium* spp.; *Penicillium* spp.; *Talaromyces* sp.; *Zygosporium* spp.) (benA) (*Aspergillus* spp.; *Penicillium* spp.)	Bt2a-F Bt2b-R (68.9 °C) Ben2f Bt2b-R (55 °C)	GGTAACCAAATCGGTGCTGCTTTC ACCCTCAGTGTAGTGACCCTTGGC TCCAGACTGGTCAGTGTGTAA ACCCTCAGTGTAGTGACCCTTGGC
Calmodulin (CaM) (Aspergillus spp.)	CMD 5 CMD 6 (55 °C)	CCGAGTACAAGGAGGCCTTC CCGATAGAGGTCATAACGTGG
Glyceraldehyde-3-phosphate dehydrogenase (GPD) (*Alternaria* spp.)	gpd1-F gpd2-R (58 °C)	CAACGGCTTCGGTCGCATTG GCCAAGCAGTTGGTTGTGC
RNA polymerase II subunit 2 (RPB2) (*Acremonium* spp.; *Penicillium* sp.; *Pichia* sp.)	fRPB2-5F fRPB2-7cR (58.5 °C)	GATGATAGAGATCATTTTGG ATGGGTAAACAAGCTATGGG
Large Subunit (D1/D2) of rRNA (*Crocicreas* spp.; *Arthrographis* sp.; *Sarocladium* sp.)	NL-1F NL-4R (55 °C)	GCATATCAATAAGCGGAGGAAAAG GGTCCGTGTTTCAAGACGG
Translation elongation factor 1-alpha (TEF1) (*Trichoderma* spp.) (*Fusarium* spp.)	EF1-728F TEF1LLEr (62 °C) ef1 ef2 (55 °C)	CATCGAGAAGTTCGAGAAGG AACTTGCAGGCAATGTGG ATGGGTAAGGARGACAAGAC GGARGTACCAGTSATCATGTT

**Table 2:** Fungal taxa found in association with *Agelas oroides*.

Taxon	Number of Isolated Strains	Genes, in Addition to ITS, Used for Molecular Identification (“-“ Indicates No Additional Genes Were Analyzed)
*Penicillium steckii*	47	Tub2
*Penicillium* sp.	12	-
*Cladosporium limoniforme*	10	ACT
*Aspergillus* sp.	8	-
*Cladosporium halotolerans*	8	ACT
*Cladosporium* sp.	8	-
*Cladosporium sphaerospermum*	8	ACT
*Penicillium brevicompactum*	8	Tub2
*Acremonium sclerotigenum*	7	RPB2
*Candida* sp.	7	-
*Parengyodontium album*	7	Tub2
*Cladosporium ramotenellum*	6	ACT
*Alternaria alternata*	5	GPD
*Aspergillus niger*	5	Cam
*Aspergillus flavus*	4	Cam
*Penicillium chrysogenum*	4	Tub2
*Penicillium citrinum*	4	Tub2
*Alternaria* sp.	3	-
*Aspergillus tubingensis*	3	Cam
*Cladosporium aciculare*	3	ACT
*Cladosporium perangustum*	3	ACT
*Gelasinospora* sp.	3	-
*Rhizopus* sp.	3	-
*Trichoderma* sp.	3	-
*Acremonium* sp.	2	-
*Aspergillus protuberus*	2	Cam
*Chaetosphaeria* sp.	2	-
*Cladosporium dominicanum*	2	ACT
*Cladosporium tenellum*	2	ACT
*Crocicreas coronatum*	2	D1/D2
*Fusarium acuminatum*	2	TEF1
*Penicillium adametzioides*	2	Tub2
*Penicillium capsulatum*	2	Tub2
*Trametes* sp.	2	-
*Trichoderma atroviride*	2	TEF1
*Trichoderma orientale*	2	TEF1
*Arthrographis kalrae*	1	D1/D2
*Aspergillus fumigatiaffinis*	1	Cam
*Aspergillus insulicola*	1	benA
*Aspergillus sydowii*	1	Cam
*Aspergillus novoparasiticus*	1	Cam
*Chaetomium longiciliata*	1	Tub2
*Cladosporium aggregatocicatricatum*	1	ACT
*Cladosporium angustisporum*	1	ACT
*Cladosporium longicatenatum*	1	ACT
*Cystidiodontia* sp.	1	-
*Exophiala* sp.	1	-
*Fusarium brachygibbosum*	1	TEF1
*Fusarium equiseti*	1	TEF1
*Microascus* sp.	1	-
*Monocillium* sp.	1	-
*Nigrospora osmanthi*	1	Tub2
*Penicillium astrolabium*	1	benA
*Penicillium coffeae*	1	Tub2
*Penicillium digitatum*	1	Tub2
*Penicillium simile*	1	benA
*Penicillium sizovae*	1	Tub2
*Penicillium wotroi*	1	Tub2
*Pichia guilliermondii*	1	RPB2
*Pichia* sp.	1	-
*Plectosphaerella* sp.	1	-
*Rhodotorula* sp.	1	-
*Sarocladium bacillisporum*	1	D1/D2
*Talaromyces funiculosus*	1	Tub2
*Trichoderma atrobrunneum*	1	TEF1
*Trichoderma gamsii*	1	TEF1
*Trichoderma guizhouense*	1	TEF1
*Zygosporium masonii*	1	Tub2, D1/D2
*Zygosporium pseudogibbum*	1	Tub2

## Data Availability

Sequence data is available at NCBI.
